# Therapeutic effects of regorafenib after sorafenib monotherapy with advanced hepatocellular carcinoma including Child–Pugh classification B: A retrospective observational study

**DOI:** 10.1097/MD.0000000000021191

**Published:** 2020-07-17

**Authors:** Satoshi Komiyama, Kazushi Numata, Katsuaki Ogushi, Satoshi Moriya, Hiroyuki Fukuda, Makoto Chuma, Shin Maeda

**Affiliations:** a Gastroenterological Center; b Chemotherapy Department, Yokohama City University Medical Center; c Department of Gastroenterology, Graduate School of Medicine, Yokohama City University, Yokohama, Kanagawa, Japan.

**Keywords:** hepatocellular carcinoma, regorafenib, sorafenib

## Abstract

The therapeutic effect of regorafenib was previously demonstrated in patients with advanced hepatocellular carcinoma (HCC) and Child–Pugh classification A (CP-A) whose disease progressed during sorafenib treatment in a phase III trial. However, treatment options are limited for patients with advanced HCC other than CP-A. In this study, we aimed to evaluate the therapeutic effect of regorafenib on advanced HCC patients including those with Child–Pugh classification B (CP-B).

We retrospectively analyzed the medical records of 21 patients with advanced HCC who were treated with regorafenib after sorafenib monotherapy at our hospital from July 2017 to April 2018 and were followed up until September 2019. Patients were classified according to liver function and adverse events experienced during sorafenib treatment and were started on regorafenib with a pre-defined reduced starting dose along with a dose reduction and schedule change based on the judgement of the attending physician.

At regorafenib initiation, 13 and 8 patients were classified as CP-A and CP-B, respectively. In all patients with CP-B, the starting dose of regorafenib was reduced, and the pre-defined starting-dose sets were applied to 17 (81%) patients. The median duration of regorafenib treatment in patients with CP-A and CP-B were 4.1 months and 2.0 months, respectively, with no significant difference. The median overall survival from regorafenib initiation (OS-r) and sorafenib initiation (OS-s) was 13.2 months and 30.9 months, respectively. In subgroup analysis, OS-r was 16.3 months in patients with CP-A and 10.1 months with CP-B with no significant difference (*P* = .44), whereas OS-r was 16.3 months in patients with modified albumin-bilirubin Grade 1/2a and 13.2 months in patients with Grade 2b, with no significant difference. There was no clear difference in the incidence rate of ≥grade 3 adverse events between CP-A and CP-B. OS-r and OS-s were significantly correlated.

Even patients with impaired liver function achieved the desired therapeutic effects by safely reducing the starting dose of regorafenib according to both impaired liver function and adverse events during pretreatment. Regorafenib may be considered to be an effective treatment after sorafenib monotherapy in patients with impaired liver function.

## 1 Introduction

According to the GLOBOCAN 2018 estimates from the International Agency for Research on Cancer, liver cancer is the sixth leading cause of morbidity and the fourth leading cause of mortality, with 840,000 new cases with poor prognosis and 780,000 deaths per year.^[1]^ According to the guidelines proposed by the American Association for the Study of Liver Diseases, treatment with molecular targeted agents is recommended for patients with advanced hepatocellular carcinoma (HCC) who exhibit portal vein invasion or distant metastasis including lymph node metastasis with Child–Pugh classification A (CP-A) or B (CP-B).^[2]^ After the SHARP trial established sorafenib as a standard treatment for advanced HCC not indicated for local treatment,^[3]^ the efficacy of multiple molecular targeted agents was investigated as first-line and second-line chemotherapy after sorafenib. As of October 2019, there are 4 molecular targeted agents approved for HCC in Japan: sorafenib, lenvatinib, regorafenib, and ramucirumab.^[4–6]^ In the RESORCE trial, a randomized, double-blind, parallel-group, phase III trial which enrolled patients with HCC in Barcelona Clinic Liver Cancer (BCLC) stage B or C and CP-A,^[4]^ the median overall survival (mOS) of the regorafenib group was 10.6 months, which was significantly better than that of the placebo group (7.8 months [hazard ratio = 0.63]). The median progression-free survival (PFS) according to modified Response Evaluation Criteria in Solid Tumors (mRECIST) was 3.1 months in the regorafenib group. The objective response rate (ORR) was 11% and the disease control rate (DCR) was 65% in the regorafenib group. In the regorafenib group, the following grade 3 and 4 treatment-emergent adverse events described according to the National Cancer Institute Common Terminology Criteria for Adverse Events version 4.0 (NCI-CTCAE v4.0) were observed in 56% and 11% of all the patients, respectively: hypertension (15%), hand-foot skin reaction (13%), fatigue (9%), diarrhea (3%), and treatment-related deaths (2%). Based on this result, the effectiveness of regorafenib as a second-line chemotherapy after sorafenib was demonstrated in patients with CP-A. However, the therapeutic effect of these molecular targeted agents was verified insufficiently in patients with CP-B. In the GIDEON study, a prospective observational study of patients with HCC treated with sorafenib including patients with CP-B, there was no obvious difference in the type and incidence of adverse events between CP-A and CP-B.^[7]^ These findings suggest that sorafenib can be administered even in patients with CP-B by appropriate patient selection and careful follow-up. Given that tolerance to sorafenib was one of the eligibility criteria for the RESORCE trial, if sorafenib is tolerated, regorafenib may be safely administered in advanced HCC patients with CP-B. Although observational study was performed on patients with HCC treated with regorafenib as second-line chemotherapy after sorafenib, few reports included patients with CP-B, while others included patients with CP-A only.^[8,9–11]^ Hence, the efficacy and safety of regorafenib after sorafenib treatment in advanced HCC patients with CP-B have not been fully clarified, and an effective treatment strategy after sorafenib monotherapy in patients with BCLC stage B and C, and CP-B is still unclear. In this study, we reported the efficacy and safety of regorafenib as second-line chemotherapy after treatment with sorafenib as well as the factors contributing to survival in patients with HCC and CP-B at our hospital.

## 2 Methods

### 2.1 Patients

We retrieved the medical records of the 22 advanced HCC patients who were started on regorafenib as second-line chemotherapy after sorafenib between July 2017 and April 2018 at our hospital, who were followed up until September 2019. The eligibility criteria included patients with unresectable or metastatic HCC that progressed on sorafenib monotherapy and those who had tolerance to sorafenib, which is defined as receiving ≥400 mg daily for at least 20 of the 28 days before discontinuation. The cause of sorafenib discontinuation included either disease progression or adverse events. The diagnosis of HCC was confirmed by pathological assessment or dynamic contrast-enhanced computed tomography (CECT) findings. The diagnostic criteria in dynamic CECT images of HCC were determined to be arterial phase “wash-in” and portal venous phase “wash-out.” We excluded a patient who was treated with sorafenib and an immune checkpoint inhibitor in clinical trial from the study. Finally, 21 advanced HCC patients were included in this retrospective study.

### 2.2 Regorafenib treatment

We evaluated liver function using Child–Pugh (CP) score and modified albumin-bilirubin, (mALBI) score before regorafenib treatment.^[12]^ In principle, the administration schedule of regorafenib was 3 weeks out of 4 weeks per cycle, and the starting dose was 160 mg/d. However, in addition to dose reduction and schedule change after starting regorafenib, we reduced the starting dose of regorafenib due to adverse events incurred during sorafenib treatment and impaired liver function at regorafenib initiation. As shown in Table 1, we assigned patients to 4 groups according to the CP classification at regorafenib initiation and adverse events during sorafenib treatment and set the starting dose for each group.

**Table 1:**
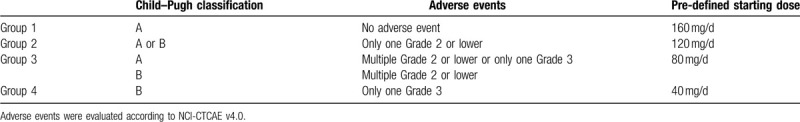
Group classification by Child–Pugh classification at regorafenib initiation and adverse events during sorafenib treatment.

### 2.3 Radiological response

Radiological response was evaluated by CECT with Response Evaluation Criteria in Solid Tumors version 1.1 (RECIST v1.1), and CECT images taken at intervals of ≥6 weeks were used in radiological response evaluation.

### 2.4 Statistical analysis

PFS was defined as the time from regorafenib initiation to the time of disease progression, as determined by RECIST v1.1, or death. OS-r was defined as overall survival on death or the date of last visit from regorafenib initiation, and OS-s was defined as overall survival on death or the date of last visit from sorafenib initiation. The proportion of patients who achieved complete response (CR) and partial response (PR) with RECIST v1.1 was defined as objective response rate (ORR). The proportion of patients who achieved CR, PR, and stable disease (SD) with RECIST v1.1 was defined as DCR. Adverse events were evaluated according to NCI-CTCAE v4.0. The Mann–Whitney *U* test was used to compare background factors in 2 groups. Survival outcomes were estimated using the Kaplan–Meier method and were compared using log rank test. The correlation between the 2 groups was evaluated using the Spearman rank correlation coefficient. A *P*-value of <.05 was considered as statistically significant. Our retrospective study was conducted with the approval of the institutional review board and in compliance with the principles of the Declaration of Helsinki, after obtaining informed consent from each of the participating patients. We also observed the applicable local laws.

## 3 Results

### 3.1 Baseline characteristics

The clinical characteristics of patients at regorafenib initiation are summarized in Table 2. First-line systemic therapy involved sorafenib administered alone with a median duration of 10 months. At regorafenib initiation, there were 13 patients (62%) with CP-A, and 8 patients (38%) with CP-B. There was one case of liver occupancy ≥50% and one case of main branch of portal vein invasion. There were 11 cases with extrahepatic metastases. Prior to regorafenib initiation, 9 patients had alpha-fetoprotein (AFP) level ≥400 ng/mL. The pre-defined starting dose of regorafenib was administered based on the classification as follows: 7 patients in Group 1, 3 patients in Group 2, 10 patients in Group 3, and 1 patient in Group 4. The actual starting doses for regorafenib were 160, 120, 80, and 40 mg/d in 6 patients (29%), 2 patients (10%), 12 patients (57%), and 1 patient (5%), respectively, and the pre-defined starting dose was administered to 17 patients (81%). In all patients with CP-B, the starting dose of regorafenib was reduced. The median duration of regorafenib treatment was 2.8 months in all 21 patients, and in patients with CP-A and CP-B was 4.1 months and 2.0 months, respectively, with no significant difference (*P* = .58). Lenvatinib was administered in 8 patients (38%) as third-line chemotherapy after regorafenib.

**Table 2:**
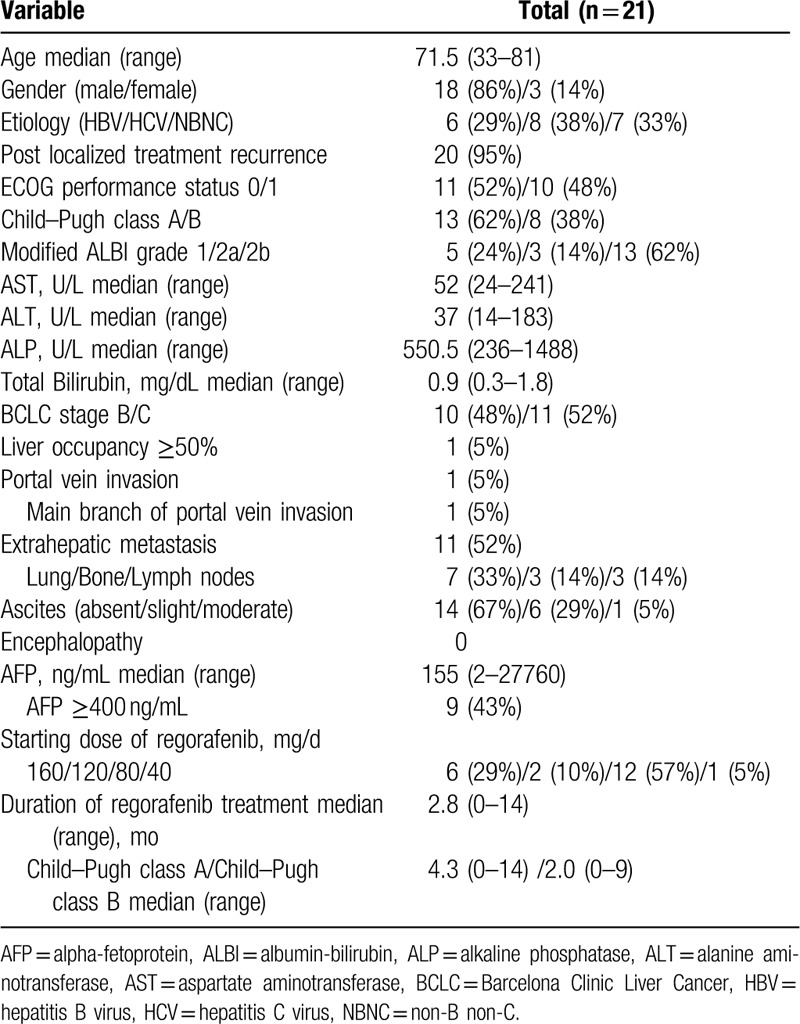
Characteristics of the patients.

### 3.2 Efficacy

**Figure 1:**
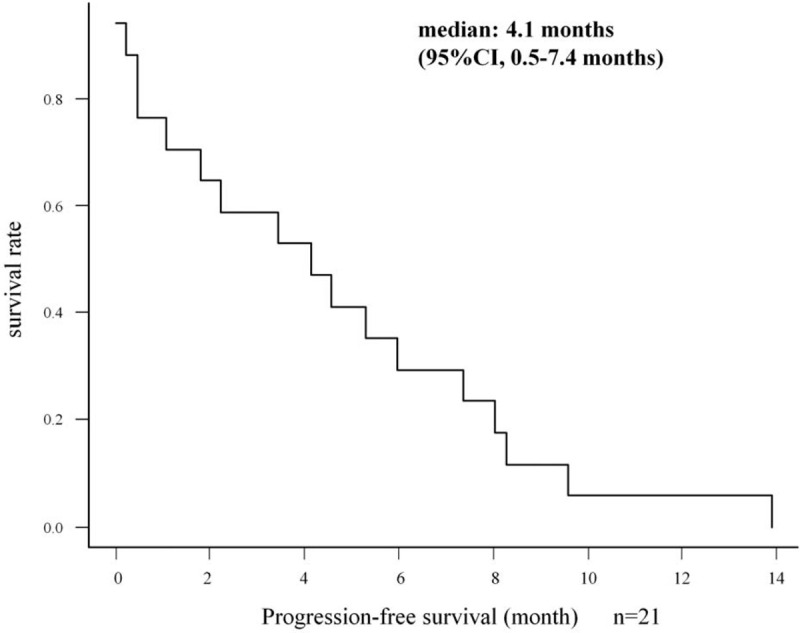
Progression-free survival in Kaplan–Meier analyses. Median progression-free survival was 4.1 months in patients with advanced hepatocellular carcinoma treated with regorafenib after sorafenib monotherapy.

**Figure 2:**
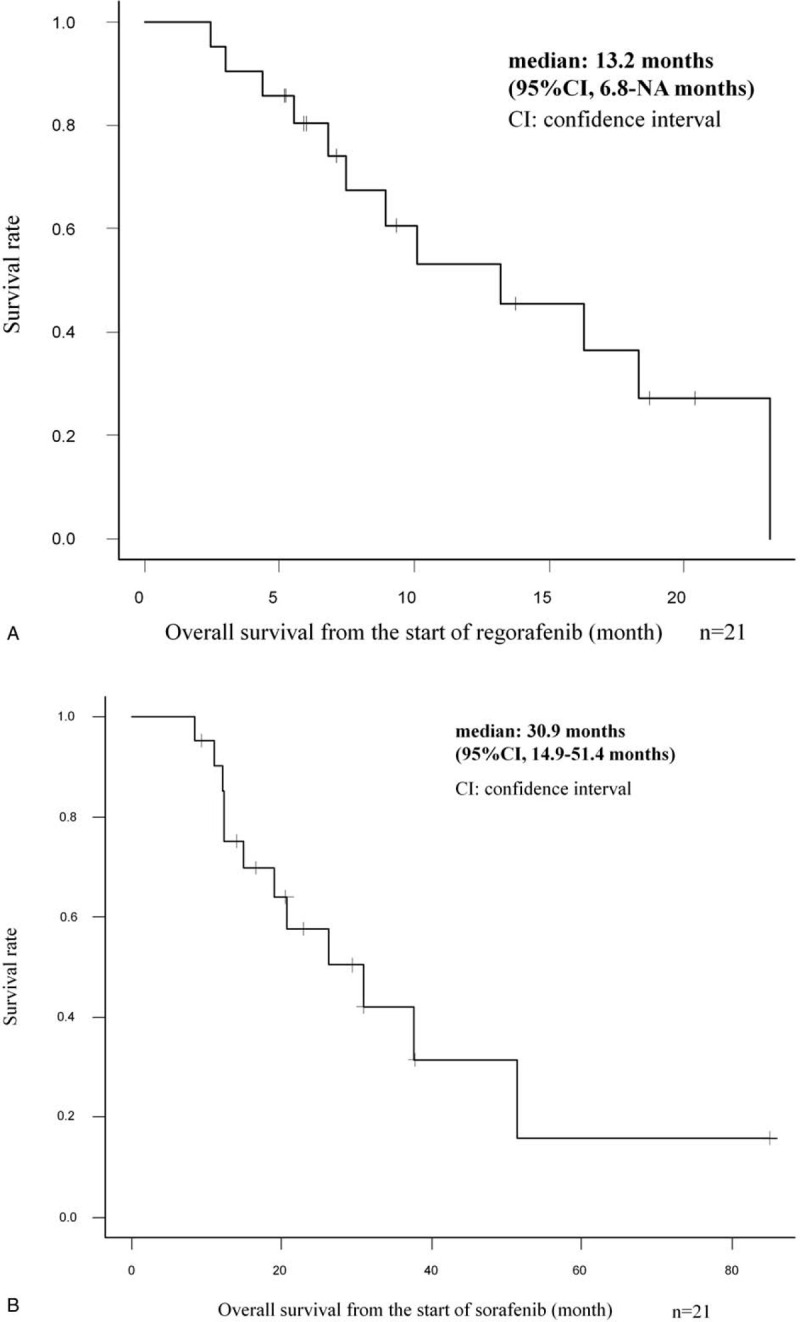
Kaplan–Meier analyses of (A) overall survival from regorafenib initiation and (B) overall survival from sorafenib initiation, representing the overall survival of patients with advanced hepatocellular carcinoma treated with regorafenib after sorafenib monotherapy.

**Figure 3:**
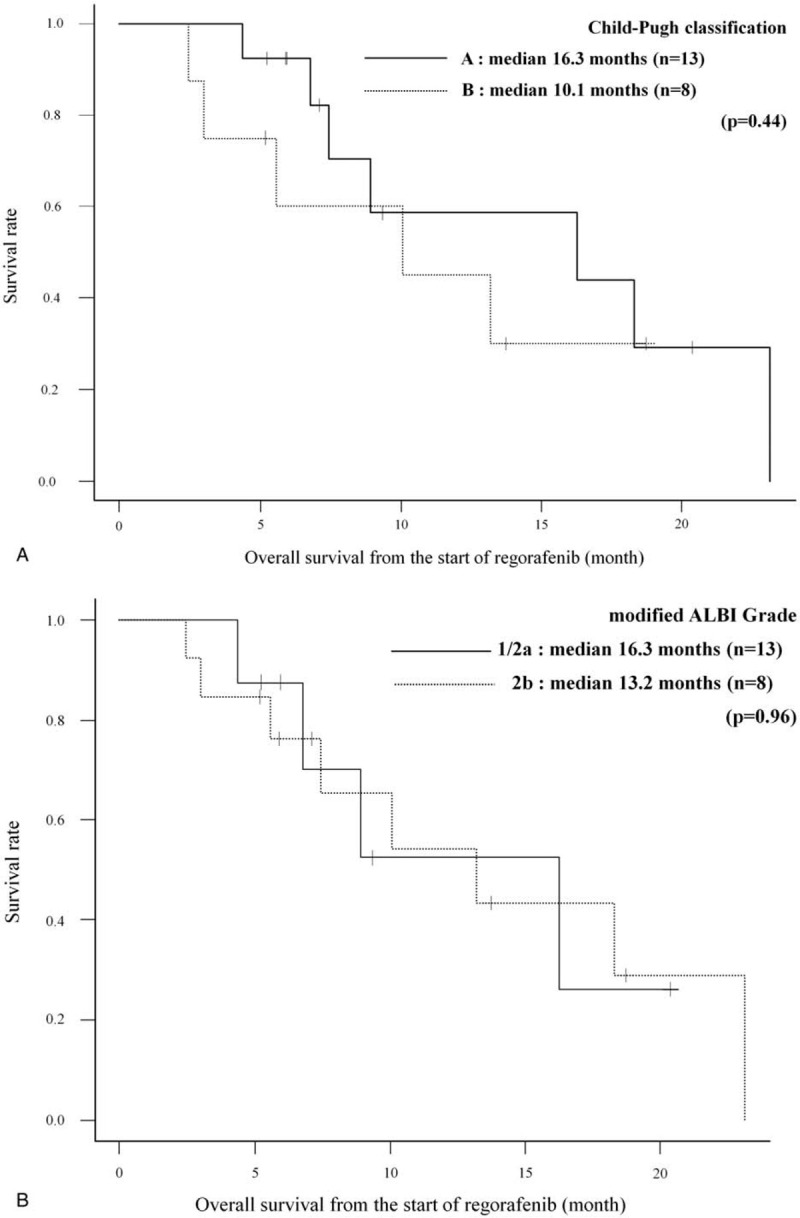
Subgroup analyses of overall survival from regorafenib initiation according to liver function. (A) Overall survival of patients with Child–Pugh A was 16.3 months and those with Child–Pugh B was 10.1 months (*P* = .44). (B) Overall survival of patients with modified albumin-bilirubin (mALBI) score 1/2a was 16.3 months and those with mALBI score 2b was 13.2 months (*P* = .96).

**Figure 4:**
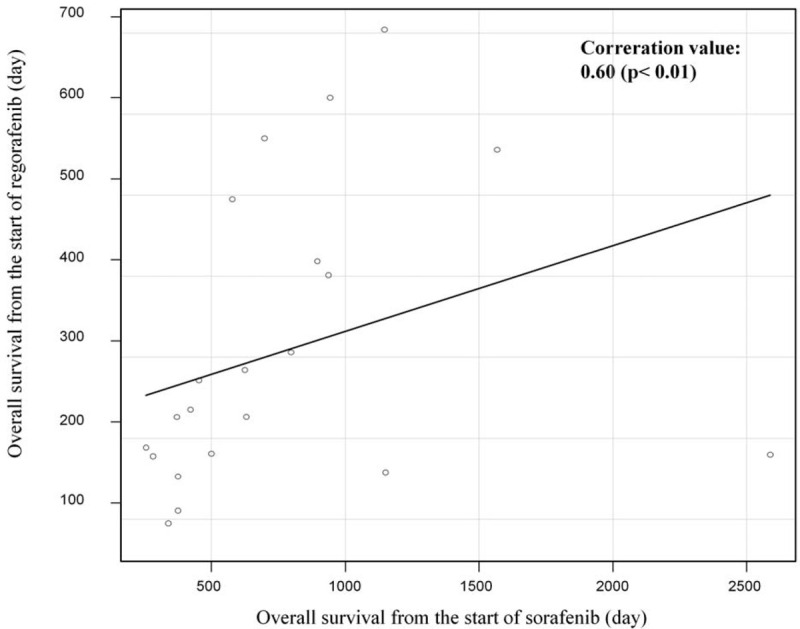
Correlation analyses of overall survival from regorafenib initiation and from sorafenib initiation. Overall survival from regorafenib and sorafenib initiation correlated with a correlation coefficient of 0.60 (*P* < .01).

In our study, there are 3 patients in whom the radiological response could not be evaluated as the best response. Of all 21 patients, 2 patients (9.5%) achieved PR, whereas none achieved CR, resulting in an RR of 9.5%. SD and progressive disease were observed in 13 (61.9%) and 3 (14.3%) patients, respectively, as the best response. In one of the patients who achieved PR, over 50% AFP decrease from baseline was observed after confirmation of PR. DCR was 71.4% (Table 3). In 21 patients, the median PFS (mPFS) was 4.1 months (95% CI, 0.5–7.4 months), the median OS-r (mOS-r) was 13.2 months (95% CI, 6.8 months–NA), and the median OS-s (mOS-s) was 30.9 months (95% CI, 14.9–51.4 months) (Figs. 1 and 2). In subgroup analysis, the mOS-r in patients with CP-A was 16.3 months (95% CI, 6.8 months–NA), and in those with CP-B, it was 10.1 months (95% CI, 2.5 months–NA), with no significant difference (*P* = .44), whereas the mOS-r in patients with mALBI Grade 1/2a was 16.3 months (95% CI, 4.4 months–NA) and in patients with mALBI Grade 2b, it was 13.2 months (95% CI, 5.6 months–NA), with no significant difference (*P* = .96) (Fig. 3). mOS-r and mOS-s were significantly correlated with a correlation coefficient of 0.60 (*P* < .05) (Fig. 4).

**Table 3:**
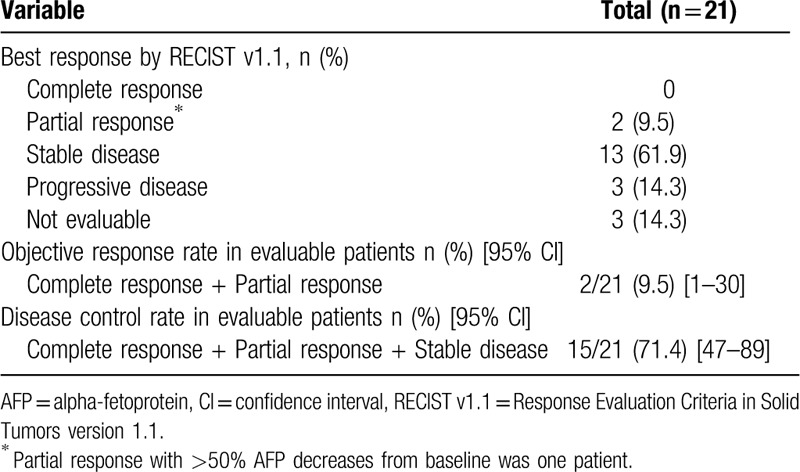
Efficacy of regorafenib treatment in patients with sorafenib alone in first-line systemic therapy.

### 3.3 Safety

Adverse events were observed in 21 patients, including fatigue (n = 14, 67%), anorexia (n = 9, 43%), hand-foot skin reaction (n = 8, 38%), diarrhea (n = 5, 24%), hoarseness (n = 4, 19%), vomiting (n = 3, 14%), peripheral neuropathy (n = 3, 14%), rash (n = 2, 10%), and transaminase elevation, mucositis, and stomatitis observed one by one (5%). Grade 3 hand-foot skin reaction was observed in 3 patients (14%), and diarrhea, fatigue, vomiting, and anorexia were observed in 1 patient (5%). In the subgroup analysis, 7 patients (33%) experienced grade 3 adverse events including 5 out of 13 (38%) patients with CP-A and 2 out of 8 (25%) patients with CP-B. There were 2 grade 3 adverse events out of 8 (25%) patients with mALBI Grade 1/2a, and 5 grade 3 adverse events out of 13 (38%) patients with mALBI Grade 2b. There was no clear difference in the frequency of grade 3 adverse events between CP-A and CP-B, mALBI Grade 1/2a and Grade 2b, and Group 1/2 and 3/4 in the classification for determining the starting dose of regorafenib. There were no treatment-associated mortalities observed. The cause of death of all those who died during follow-up was the primary disease.

## 4 Discussion

In this study, we performed a retrospective analysis of patients with advanced HCC to whom regorafenib was administered as second-line chemotherapy after sorafenib monotherapy, including patients with CP-B in clinical practice. In addition to the dose reduction and the schedule change after starting treatment, the patients were classified into 4 groups based on liver function and the adverse events that occurred during sorafenib treatment; the starting doses of regorafenib were set for each group. The pre-defined starting dose sets were administered to 17 (81%) patients, and resulted in similar survival benefit and incidence rate of ≥grade 3 adverse events in patients with HCC and CP-A and CP-B. In addition, OS-r and OS-s were significantly correlated.

In the RESORCE trial, only patients with CP-A were included.^[4]^ Although there are many patients with advanced HCC with CP-B after sorafenib, the therapeutic effect of regorafenib has been insufficiently verified in these patients. In the analysis of long-term survivors receiving sorafenib for advanced HCC, the long-term continuation of sorafenib was reported as a good prognostic factors, and some of the long-term survivors started sorafenib with reduced starting doses, depending on their individual clinical characteristics.^[13]^ In addition, in regorafenib treatment for gastrointestinal stromal tumor and colorectal cancer, treatment could be continued while suppressing the relapse or exacerbation of adverse events by dose reduction or a change in the administration schedule based on the adverse events occurring during prior treatment.^[14,15]^ This suggested that reductions in the starting dose of regorafenib based on the clinical characteristics of patients with advanced HCC improved their prognosis. We utilized the liver function and adverse events during sorafenib treatment as the deciding criteria in patients to determine the starting dose of regorafenib. In the GIDEON study, there was no significant difference in the starting dose of sorafenib between patients with CP-A and CP-B, resulting in equivalent safety profiles of sorafenib, and adverse events leading to treatment discontinuation were more common in CP-B than in CP-A.^[7]^ Hence, we classified patients into 4 groups, mainly according to adverse events during sorafenib administration; each group received a reduced starting dose along with dose reduction and schedule change after starting regorafenib. In addition, the starting dose of regorafenib in patients with CP-B was further reduced if a grade 3 adverse event occurred during sorafenib administration. As a result, the pre-defined starting dose-sets were applied to 17 (81%) of the 21 patients and survival and incidence rates of adverse events equivalent to those of the RESORCE study were obtained in our study. Moreover, in the subgroup analysis, mOS was comparable in patients with CP-A and CP-B, as well as patients with mALBI Grade 1/2a and Grade 2b prior to regorafenib treatment. In tyrosine kinase inhibitor (TKI) treatment for unresectable HCC, it has been reported that the prognosis of patients with mALBI Grade 1/2a was significantly better than that of patients with mALBI Grade 2b when liver function was compared before initiation.^[16]^ Although only a small number of patients were examined, it was suggested that a reduction in the starting dose of regorafenib led to a good therapeutic effect in patients with poor liver function of CP-B or mALBI Grade 2b. In addition, there was no significant difference in the incidence of severe adverse events between CP classification and groups in the classification for determining the starting dose of regorafenib, which showed that a reduction in the starting dose of regorafenib may contribute to a reduction in severe adverse events in patients with liver dysfunction.

In an observational study conducted in Japan on regorafenib-treated patients after refractory or intolerant to sorafenib monotherapy, mPFS and mOS after regorafenib initiation were reported to be 6.9 and 17.3 months, respectively.^[8]^ Compared with our study, mPFS and mOS were better, but the proportion of CP-B patients was 9% in this study and 38% in our study. Therefore, it was thought that the prognostic difference was caused by the inclusion of more patients with liver dysfunction. In contrast, an observational study of patients in which regorafenib was administered after sorafenib failed, including CP-B and CP-C in 10% of all patients in South Korea, reported mPFS as 3.7 months, which was worse than that observed in our study (4.1 months), and the 1-year overall survival rate was 54.6% with mOS not reached at the time of analysis.^[17]^ Compared with the median duration of regorafenib treatment in this study (2.4 months), that in our study (2.8 months) was slightly longer, despite a higher proportion of CP-B patients (38%). Long-term continuation of regorafenib treatment owing to reduction in starting dose of regorafenib based on the clinical characteristics of patients may explain the difference in mPFS between these studies. In addition, 8 patients (38%) were treated with lenvatinib as third-line chemotherapy after regorafenib in our study, whereas in South Korea observational studies, other systemic chemotherapy was used, so the results of the additional analysis on survival time of this study are expected. Lenvatinib was shown to be effective as a first-line chemotherapy for unresectable advanced HCC in the REFLECT trial,^[5]^ and it is reported that treatment response and incidence rate of adverse events after lenvatinib were similar, irrespective of past TKI therapy.^[18]^

Of the drugs that have been approved in Japan as molecular targeted agents for unresectable HCC, regorafenib and ramucirumab have shown efficacy in phase III trials as a second-line chemotherapy after sorafenib and regorafenib is often administered because ramucirumab is indicated only for patients with serum AFP values of 400 ng/mL or higher. In our study, mOS from sorafenib initiation correlated with mOS after regorafenib initiation. Previous studies have reported that survival time after sorafenib initiation is correlated with the survival time after failed sorafenib treatment in advanced HCC.^[19,20]^ This suggested the importance of second-line treatment choices after sorafenib, indicating the validity of regorafenib as a treatment of choice.

There are some limitations to this study. First, this was a retrospective study at a single center; as such, there may be unintentional selection bias. Second, the adjustment of the starting dose of regorafenib is a matter of judgment of the individual physician. Therefore, it is necessary to establish a standard practice for reducing the starting dose of regorafenib based on the pretreatment liver function and adverse events that occurred in prior treatment, and to verify its therapeutic effect through prospective studies.

## 5 Conclusion

In our study, we retrospectively analyzed patients with advanced HCC in whom regorafenib was administered as second-line chemotherapy after sorafenib including patients with poor liver function of CP-B or mALBI Grade 2b. It is suggested that even advanced HCC patients with impaired liver function achieved good therapeutic effects while maintaining safety by reducing the starting dose of regorafenib according to the decreased liver function and adverse events during sorafenib treatment, which indicated that regorafenib may be effective as a treatment of choice after sorafenib treatment even in advanced HCC patients with impaired liver function.

## Acknowledgments

The authors would like to thank Editage (www.editage.com) for English language editing.

## Author contributions

Satoshi Komiyama, Kazushi Numata, Katsuaki Ogushi, Satoshi Moriya, Hiroyuki Fukuda, and Makoto Chuma reviewed the literature and drafted the manuscript; Maeda S were responsible for the revision of the manuscript for important intellectual content; all authors issued final approval for the version to be submitted.
